# Macroscopically Anisotropic Structures Produced by Light-induced Solvothermal Assembly of Porphyrin Dimers

**DOI:** 10.1038/s41598-018-28311-2

**Published:** 2018-07-23

**Authors:** Yasuyuki Yamamoto, Yushi Nishimura, Shiho Tokonami, Norihito Fukui, Takayuki Tanaka, Atsuhiro Osuka, Hideki Yorimitsu, Takuya Iida

**Affiliations:** 1 Department of Physics, Graduate School of Science, Osaka Prefecture University, 1-1 Gakuencho, Naka-ku, Sakai, Osaka, 599-8531 Japan; 2 Research Institute for Light-induced Acceleration System (RILACS), Osaka Prefecture University, 1-1 Gakuencho, Naka-ku, Sakai, Osaka, 599-8531 Japan; 3 Department of Applied Chemistry, Graduate School of Engineering, Osaka Prefecture University, 1-1 Gakuencho, Naka-ku, Sakai, Osaka, 599-8531 Japan; 4 Department of Chemistry, Graduate School of Science, Kyoto University, Sakyo-ku Kyoto, 606-8502 Japan; 5 Present Address: Division of Molecular Materials Science, Graduate School of Science Osaka City University, Sumiyoshi-ku Osaka, 558-8585 Japan; 6 Present Address: Department of Molecular and Macromolecular Chemistry, Graduate School of Engineering, Nagoya University, Nagoya, 464-8603 Japan

## Abstract

Porphyrin-based molecules play an important role in natural biological systems such as photosynthetic antennae and haemoglobin. Recent organic chemistry provides artificial porphyrin-based molecules having unique electronic and optical properties, which leads to wide applications in material science. Here, we successfully produced many macroscopically anisotropic structures consisting of porphyrin dimers by light-induced solvothermal assembly with smooth evaporation in a confined volatile organic solvent. Light-induced fluid flow around a bubble on a gold nanofilm generated a sub-millimetre radial assembly of the tens-micrometre-sized petal-like structures. The optical properties of the petal-like structures depend on the relative angle between their growth direction and light polarisation, as confirmed by UV-visible extinction and the Raman scattering spectroscopy analyses, being dramatically different from those of structures obtained by natural drying. Thus, our findings pave the way to the production of structures and polycrystals with unique characteristics from various organic molecules.

## Introduction

Natural photosynthetic systems found in plants and bacteria convert solar energy into chemical energy with 100% quantum efficiency, using nanoscale petal-like antennae comprising porphyrin-based molecules (PBMs) to harvest photons of various wavelengths^1,2^. Another example is represented by a PBM-Fe^2+^ complex found in haemoglobin, which is responsible for the delivery of oxygen by animal blood cells^3,4^. The unique properties of PBMs have been utilised in molecular electronics components^5–7^, gas sensors^8,9^, dye-sensitised solar cells^10,11^, and contrast agents for bio-imaging^12,13^, with recent advances in organic chemistry enabling syntheses of various types of porphyrin dimers, polymers, or conjugated tapes with controllable length featuring strong infrared absorption, two-photon absorption, and unique electronic properties^7,14,15^. Importantly, structurally planar porphyrins can be used to construct diversely shaped nanoscale self-assembled structures such as nanoparticles, nanosheets, and nanorods^16–20^, with morphology control playing a vital role in the development of novel porphyrin-based materials.

Remote control of molecular assembly by external field–induced modulation of the growth environment would allow the production of unconventional structures with an extended application range. For example, light-induced forces arising from electromagnetic interaction between light and matter were used to selectively assemble an artificial light-harvesting structure using metallic nanorods extracted from diversely shaped nanoparticles under resonant laser illumination^21^, and also applied to amino acid crystallisation and sulfathiazole nucleation at the air-liquid interface^22,23^. Another approach to assemblies of nano- and micron-sized objects (e.g., nanoparticles, soft oxometalates, carbon nanotubes, metallic nanoparticles, and bacteria) is based on light-induced fluid flow originating from thermofluid dynamics under heating by laser light (i.e., photothermal assembly (PTA))^24–29^. Although the above reports on PTA utilised aqueous solutions with low evaporation rates, the use of volatile organic solvents is expected to achieve faster light-induced fluid flow due to their high evaporation rate.

Here, being inspired by natural PBM-containing functional structures, we produced PBM-based structures as unconventional optical materials by utilising a high-temperature and high-evaporation-rate solvothermal process in an organic solvent under PTA conditions, since such processes are often used to prepare highly crystalline inorganic structures^30,31^. A porphyrin dimer (meso-meso, β-β, β-β triply linked Zn^II^-diporphyrin^32,33^ exhibiting exquisite crystallinity due to its planar molecular framework) was utilised as the model PBM. A toluene solution of diporphyrin was placed on a gold nanofilm and the solution/nanofilm interface was vertically illuminated with an infrared continuous wave (CW) laser to generate fluid flow for transporting and a bubble for the deposition of the transported molecules. Then, we investigated the optical properties and molecular arrangement of the prepared structures by utilising UV-visible and Raman scattering spectroscopy analyses.

## Light-induced solvothermal assembly (LSTA) of diporphyrin

**Figure 1:**
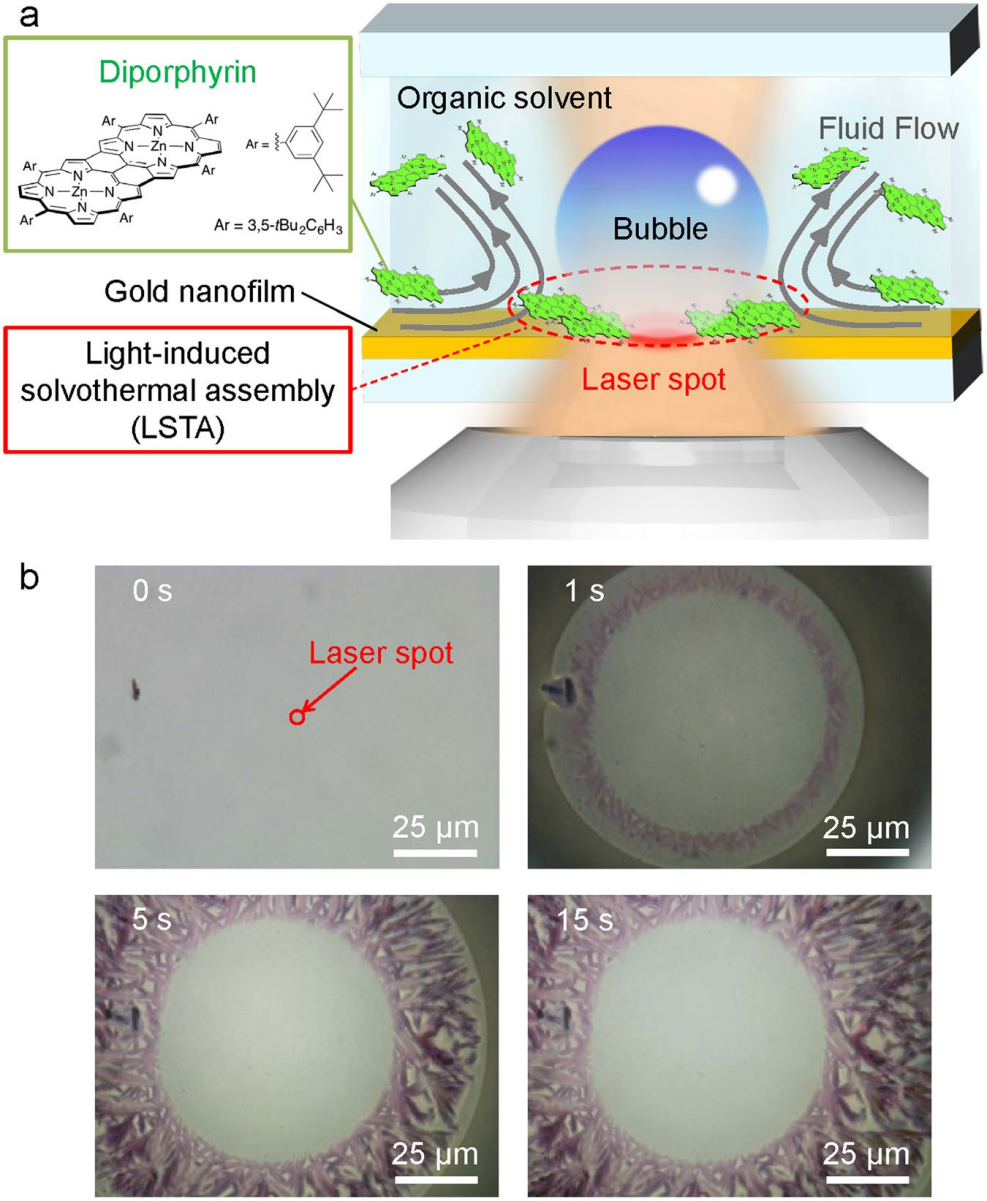
Light-induced solvothermal assembly (LSTA) of diporphyrins into anisotropic structures with petal-like shapes. (**a**) Schematic representation of LSTA. (**b**) Optical transmission images reflecting the growth of anisotropic LSTA-produced structures of diporphyrins. Numbers in images correspond to illumination times.

CW laser illumination on the gold nanofilm resulted in intense local heating, since the thermal conductivity of metal films decreases with their decreasing thickness^34,35^, inducing fluid flow and generating a sub-millimetre-sized bubble as shown in Fig. 1a (the detail of the experimental setup is in Supplementary Figure S1). The temperature of the solvent (toluene) surrounding the bubble sharply increased beyond its boiling point (111 °C), and vapourisation of the solvent afforded a supersaturated solution around the spreading bubble and subsequently produced a sub-millimetre radial assembly of many structures with petal-like shapes (Fig. 1b and Supplementary Movie S1). Notably, the bubble formation was observed already after 1-second illumination (Fig. 1b, top right), with the bright area in the centre of the above image corresponding to the region where the bubble attaches itself to the substrate, and the dark area representing the shadow of this bubble. Careful observation of the location of LSTA-produced structures in Fig. 1 shows that they are generated not at the periphery of the bubble but at the ground contact portion of the bubble and the substrate. As a hypothesis, it is considered that these structures were produced under a solvent-thermal synthesis in the diporphyrin solution slightly remaining between the bubble and the substrate under a high pressure environment due to strong pressure by bubbles and laser heating. Therefore, we call this generation method “Light-induced solvothermal assembly (LSTA)”.

**Figure 2:**
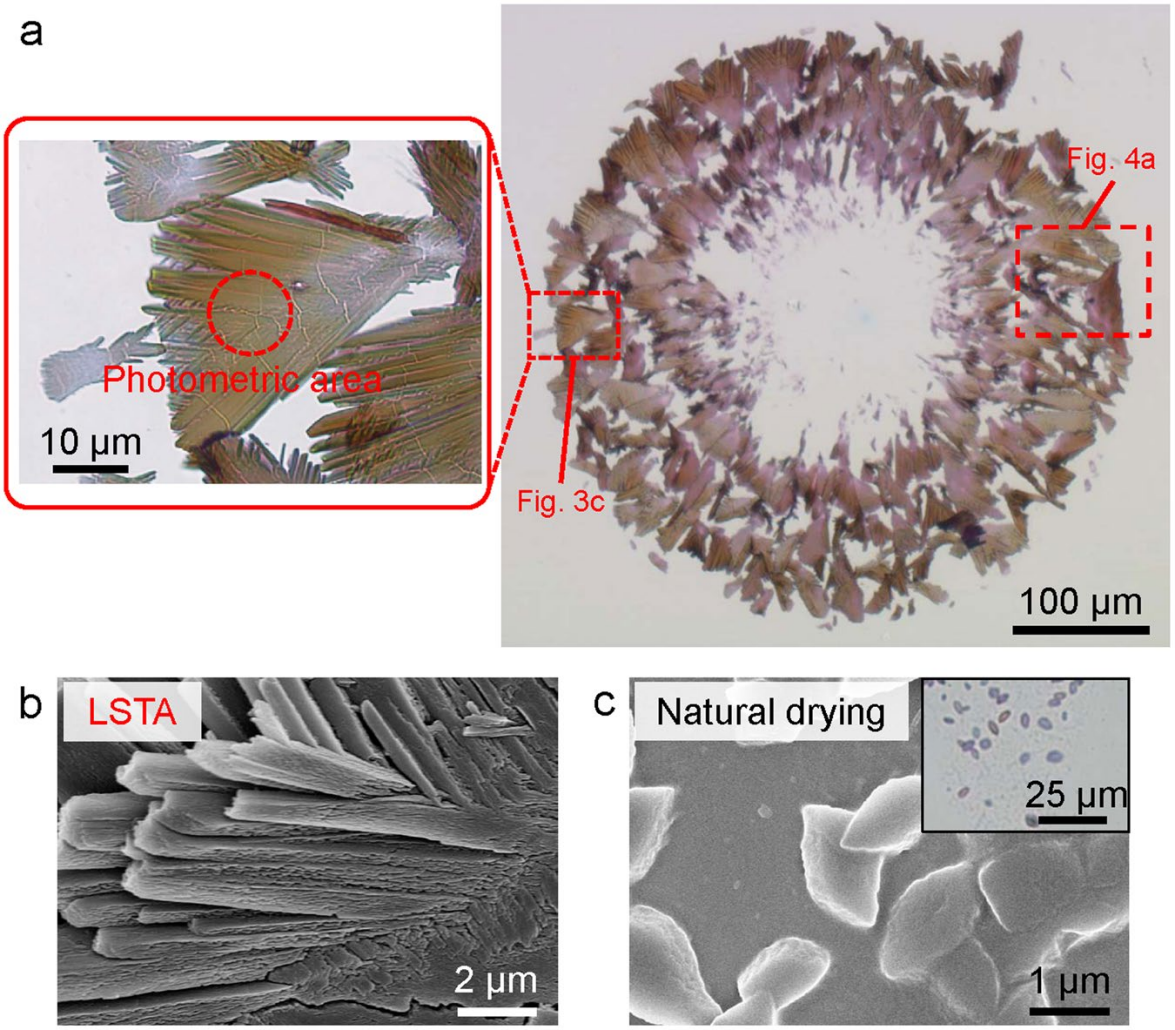
Microscopic images of diporphyrin-based structures. (**a**) Optical transmission images of LSTA-produced structures under non-polarised white light (halogen lamp). (**b**) Field emission scanning electron microscope (FE-SEM) images of an LSTA-produced petal-like structure and (**c**) of structures obtained by natural drying with an inset of the corresponding optical transmission image. Optical response dependent on polarisation angle

After the CW laser illumination was turned off, the bubble separated itself from the substrate and floated away, with the petal-like structures of diporphyrins re-dissolving unless the solvent was immediately removed. Thus, to hinder the re-dissolution, the solvent was removed with a paper wiper due to the action of capillary forces, which resulted in the irreversible formation of an assembly of tens-micrometre-sized LSTA-produced structures of diporphyrins (Fig. 2a). It has been confirmed that a part of LSTA-produced structures is separated from the substrate or changes its direction during the solvent removal process. Furthermore, field emission scanning electron microscopy (FE-SEM) analyses revealed that each petal-like structure has radially branched morphology and that structures obtained by natural drying of a toluene solution of the diporphyrin take differently rugby-ball-like morphology (Fig. 2b,c, respectively).

**Figure 3:**
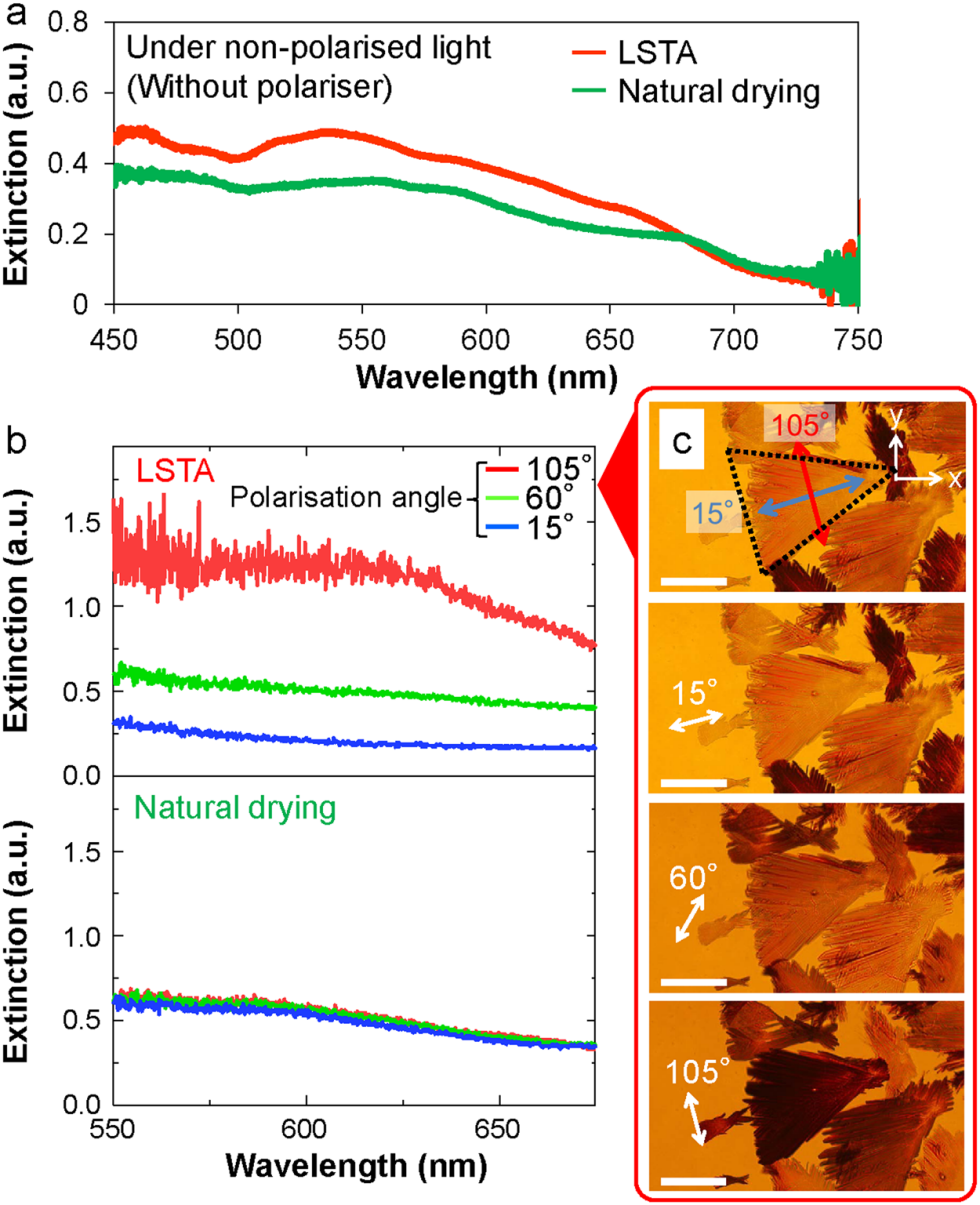
Optical anisotropy of LSTA-produced structures. (**a**) Locally observed extinction spectra of LSTA-produced structures and natural drying-produced structures (a reference spectrum of the parent diporphyrin solution is shown in Supplementary Figure S3). Both of the extinction spectra were averaged at three different positions. (**b**) Extinction spectra of an LSTA-produced structure and natural drying-produced structures recorded under various polarisation conditions. (**c**) Optical transmission images of LSTA-produced structures recorded under various polarisation conditions of white light. The length of the scale bars is 20 µm. In the top figure, the shape of a LSTA-produced structure was approximated with a triangle, and shows the polarisation angles for lowest and highest extinction.

The extinction spectra (sum of absorption and scattering) of LSTA-produced structures and natural drying-produced structures (Fig. 3a) were locally observed in the photometric area as shown in Fig. 2a, they were compared with the extinction spectrum obtained in the toluene solution of the parent diporphyrin **(**Supplementary Figure S2 and S3). Peaks around wavelengths = 458, 485 and 585 nm were not shifted, but it was confirmed that peaks around wavelength = 556, 641 nm (solution) were shifted to short wavelength region (blue shift) and long wavelength region (red shift) respectively (Supplementary Table S1). This peak shift may be due to the formation of J aggregates (blue shift) and H aggregates (red shift) in which molecules are aggregated in one dimension. Therefore, both J-aggregate and H-aggregate are considered to be contained in LSTA-produced structures and natural drying-produced structures (the stability of diporphyrin is high and it is reported that the structure is maintained even at high temperature of 500 K or more^36^, and diporphyrin degeneration by LSTA did not occur.).

The radial growth pattern of LSTA-produced structures implied that growth occurred in a specific direction and could thus result in optical anisotropic properties. To test this assumption, we measured extinction spectra of the LSTA-produced structures (Fig. 3b) and observed transmission images of them (Fig. 3c and Supplementary Movie S2) under polarised light (polarisation angle (15–105°, step = 45°)) using a polarising plate**)**. Each of the LSTA-produced structures clearly exhibited macroscopically and optically anisotropic properties. When we carefully checked the relationship between polarisation transmitted images and polarisation angles, we noticed that the morphology of the LSTA-produced structure is correlated to polarisation angle where extinction occurred most strongly in the optical transmission process. Interestingly, an LSTA-produced structure absorbed or scattered light polarised perpendicularly to the radial growth direction, being transparent to light polarised parallel to the above direction in Fig. 3c. Similar behaviour was also observed for other LSTA-produced structures, implying that these structures comprised anisotropic crystals. Conversely, a single natural drying-produced structure with rugby-ball-like shape did not exhibit pronounced anisotropic properties(Supplementary Figure S4), and showed weakly polarisation-dependent property, with this anisotropy vanishing in the averaged spectrum (Fig. 3b). These results suggest that light-induced fluid flow would transport constituent molecules around a bubble to result in a directional assembly in the case of LSTA, whereas the solution was evaporated uniformly and slowly to result in a directionless growth in the case of natural drying.

## Raman scattering properties

**Figure 4:**
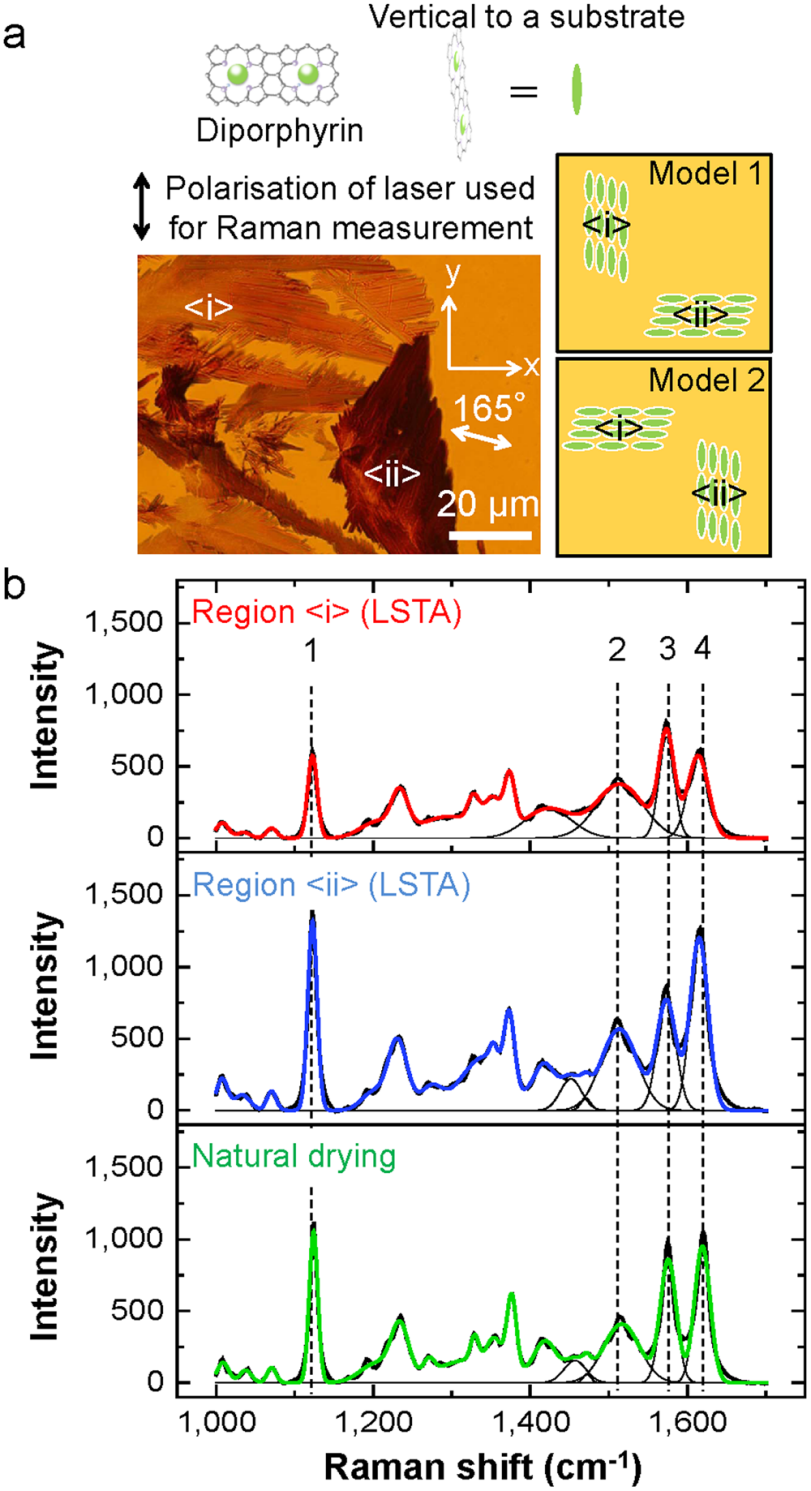
Raman scattering spectra of LSTA-produced structures and natural drying- produced structures. (**a**) Optical transmission image of LSTA-produced structures for white light polarisation angle 165° (enlarged image of a part of Fig. 2a), and models for available arrangement of diporphyrins in Region <i> and <ii> . (**b**) Raman scattering spectra of Region <i> and Region <ii> in each LSTA-produced structure averaged at five different positions in both cases. Also, Raman scattering spectrum of natural drying–produced structures averaged over five different structures is shown together. Each dotted line and number indicates the peak position of 1: C(meso)-C(Ar), Ar, 2, 3, 4: C(α)-C(meso), C(α)-C(β), C(β)-C(β), C(α)-N^37^.

The possible arranged structures of diporphyrin are considered to be the sequence shown in Fig. 4a (Model 1 and Model 2) and the sequence in which diporphyrin exists parallel to the substrate. The latter model can be rejected since it is unlikely that extinction spectra vary greatly with the polarisation angle of the illumination light as shown in Fig. 3b. Therefore, in order to investigate which array structure is possible in Fig. 4a, the Raman scattering intensities of molecular vibration modes (C (α) -C (meso), C (α) -C (β), C (β) -C (β), C (α) -N) are compared. The LSTA-produced structures showing different contrast (Region <i> and Region <ii> shown in Fig. 4a), were measured by Raman scattering spectroscopy to investigate the arrangement of diporphyrins in LSTA-produced structures. Here, the LSTA-produced structure of Region <ii> is considered to indicate a colour different from that of Region <i> because its orientation changed during solvent removal process. Fig. 4b shows the Raman scattering spectra of the Regions <i> and <ii> as well as that of natural drying-produced structure. Since the intensity at each peak wavenumber 1515 cm^−1^, 1615 cm^−1^ (C(α)-C(meso), C(α)-C(β), C(β)-C(β), C(α)-N) is higher in the order of Region <ii>, natural drying, and Region <i> (Supplementary Table S2), Model 2 is considered to be valid (Supplementary Table S2). On the other hand, at 1575 cm^-1^, the Raman intensity of Region <i> in of LSTA-produced structure showed the maximum intensity and that of natural drying-produced structure was higher than that of Region <ii>. This indicates that the π-bond at around the peak wave number of 1575 cm ^-1^, and the assumption that there is a contribution is consistent with Model 2.These results strongly support that each LSTA-produced structure exhibited the high anisotropy, and that the molecular arrangements can be modulated by LSTA, differently from isotropic natural drying-produced structures.

## Conclusions

We have demonstrated that LSTA produces macroscopically anisotropic petal-like structures of diporphyrin in an organic solvent via light-induced evaporation process, with the polarisation-dependent optical extinction of these structures being in contrast to the behaviour of natural drying-produced structures. Moreover, from the extinction spectrum and Raman spectral analysis, the following two models of diporphyrin sequence and binding were suggested: (i) an LSTA-produced structure contain both the J aggregates and H aggregates, (ii) Diporphyrin has an arrangement perpendicular to the substrate and with its planar surface oriented toward the laser illumination point. Thus, broadband anisotropic optical extinction of assembled PBMs can be used to produce micron-scale white light filters for optical interconnections comprising photonic structures, with the obtained results paving the way to laser-mediated solvothermal assembly of arbitrary nanoscale organic molecules into unconventional crystal polymorphs at the desired position on the substrate. Further measurements of electrical conductivity and other nonlinear optical responses of assembled molecules are expected to extend the applications of the above structures to nanoelectronics, nanophotonics, and next-generation bio-inspired photonic information technologies.

## Methods

### Synthesis of diporphyrin

The diporphyrin (porphyrin dimer) used in this work was prepared and characterised elsewhere^32,33^.

### Experimental setup for LSTA

Supplementary Figure S1 shows schematic illustrations of the experimental setup for LSTA. The sample holder comprising a slide glass and three cover slips (with one of them covered by a gold nanofilm) with double-sided tape (0.1-mm-thick) is shown in Supplementary Figure S1. After injection of the liquid sample into the holder, it was set on the stage of an inverted microscope (Eclipse Ti-U, Nikon, Japan) equipped with a 1064-nm CW laser light source (Supplementary Figure S1). The diameter of the laser spot was set to 1.0 μm by using a 100 × objective (Numerical Aperture = 1.3), with the power of laser light penetrating the cover slip measured by a power meter (UP17P-6S-H5 with tuner, Gentec Electro-Optics, Canada) and used throughout the experiments. The ~10-nm-thick (as measured by a profilometer (Dektak150, Takaoshin, Japan)) gold nanofilm was deposited on a cover slip as a light-absorbing layer by sputtering (E-1010, Hitachi, Japan).

After the sample holder was placed onto the stage of the inverted microscope, a diporphyrin solution in toluene (1.0 mg of diporphyrin powder was dispersed in 10 mL of toluene) was placed on the gold nanofilm and illuminated by CW laser light at an intensity of ~1.3 × 10^7^ W/cm^2^ for 3 min. During illumination, the deposition process was monitored using a cooled charge-coupled device camera (DS-Filc-L3, Nikon, Japan) under bright-field conditions. When laser illumination was turned off, the solvent was immediately removed by the capillary force of a sheet of KIMWIPES (Kimberly Clark Corp., USA).

### Microscopic observations and optical measurements

Double-sided tape was removed from the cover slip coated by the gold nanofilm to allow FE-SEM imaging (SU8010, Hitachi High-Tech., Japan) of the LSTA-produced structures. The optical extinction spectra of LSTA-produced structures were locally observed within a 10-μm-diameter photometric area by UV-visible spectrometry (USB-4000; Ocean Optics, USA), and Raman scattering spectra were acquired using a laser Raman microscope (Raman-11, Nanophoton, Japan) using 532 nm laser with *y*-polarisation. Polarisation spectroscopy was performed by incorporating a polariser (SIGMA KOKI, Japan) into the illumination system. The extinction spectrum and the Raman spectrum were analyzed using the commercial soft ware (Origin, OriginLab Corporation, USA). The natural drying–produced structures obtained after approximately 10 min. by naturally evaporating a toluene solution of the diporphyrin at room temperature (25 °C) on a substrate (gold nanofilm on a cover slip) (referred as “natural drying” in the main text) were also characterised using the same equipment.

## Electronic supplementary material

Supplementary Information Text and Figures

Supplementary Movie S1

Supplementary Movie S2
