# Supplementary material for: Macroscopically Anisotropic Structures Produced by Light-induced Solvothermal Assembly of Porphyrin Dimers
Source: Sci Rep. 2018 Jul 23;8:11108. doi: 10.1038/s41598-018-28311-2 (PMC6056561; doi:10.1038/s41598-018-28311-2)
Supplement: Supplementary file 1 — Supplementary Information Text and Figures [file 41598_2018_28311_MOESM1_ESM.pdf]

SUPPLEMENTARY INFORMATION

Macroscopically Anisotropic Structures Produced by Light-induced Solvothermal Assembly of Porphyrin Dimers

Yasuyuki Yamamoto[1,2,3], Yushi Nishimura[1,2,3,†], Shiho Tokonami[2,3,*], Norihito Fukui[4,‡], Takayuki Tanaka[4], Atsuhiro Osuka[4], Hideki Yorimitsu[4,*], Takuya Iida[1,2,*]

[1]Department of Physics, Graduate School of Science, Osaka Prefecture University,

[2]Research Institute for Light-induced Acceleration System (RILACS), Osaka Prefecture University,

[3]Department of Applied Chemistry, Graduate School of Engineering, Osaka Prefecture University, 1-1 Gakuencho, Naka-ku, Sakai, Osaka 599-8531, Japan.

[4]Department of Chemistry, Graduate School of Science, Kyoto University, Sakyo-ku, Kyoto 606-8502, Japan.

[†]Present address: Division of Molecular Materials Science, Graduate School of Science, Osaka City University, Sumiyoshi-ku, Osaka, 558-8585, Japan

[‡]Present address: Department of Molecular and Macromolecular Chemistry, Graduate School of Engineering, Nagoya University, Nagoya 464-8603, Japan

*Correspondence to: t-iida@p.s.osakafu-u.ac.jp (T. I.); yori@kuchem.kyoto-u.ac.jp (H. Y.); tokonami@chem.osakafu-u.ac.jp (S. T.)

Movie Legends:

Please see also the detail in the main text.

Supplementary Movie S1. Process of light-induced solvothermal assembly (LSTA) of the diporphyrins recorded as an optical transmission image (real time). Laser illumination started at 5 seconds after the start of movie (corresponding to **Figure 1b**).

Supplementary Movie S2. Optical transmission images of the LSTA-produced structures of diporphyrins under various polarisation angles. Polarisation angles in this movie correspond to those in **Figure 3c**.

Supplementary Figures and Tables:

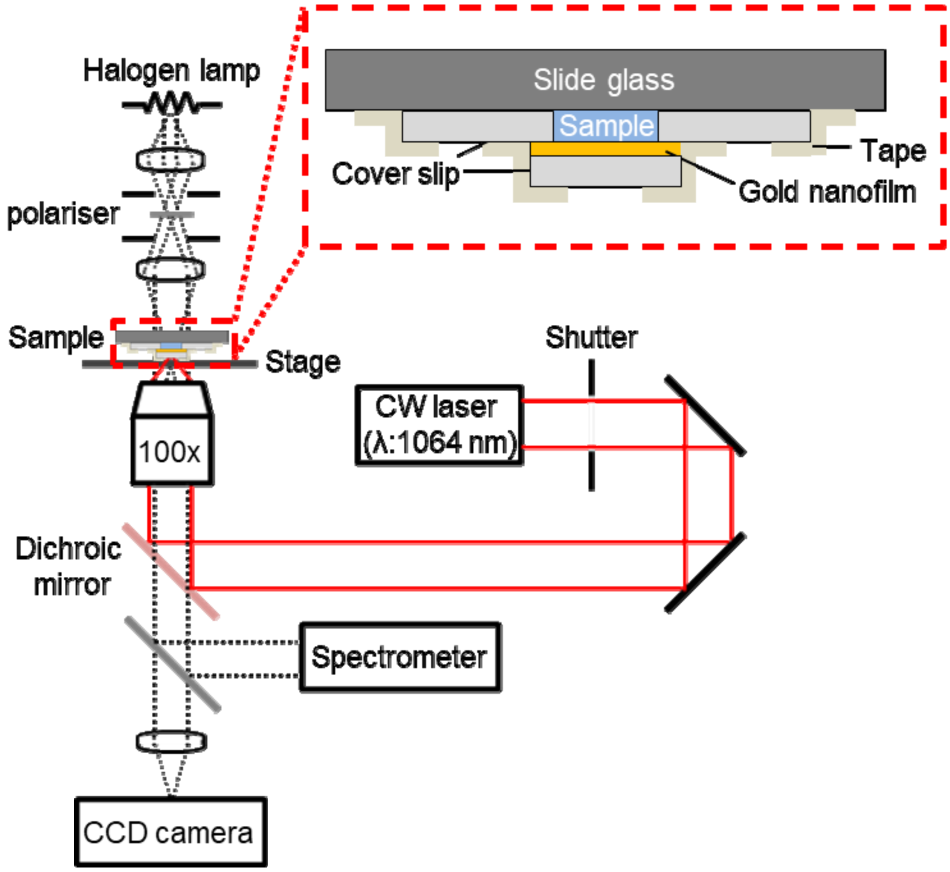

Supplementary Figure S1 | Schematic illustrations of the experimental setup for LSTA.

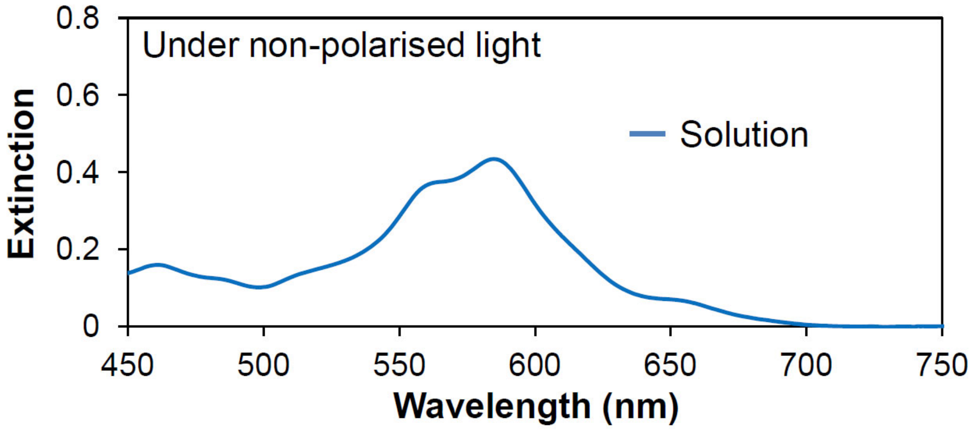

Supplementary Figure S2 | Extinction spectrum of diporphyrin dispersed in toluene.

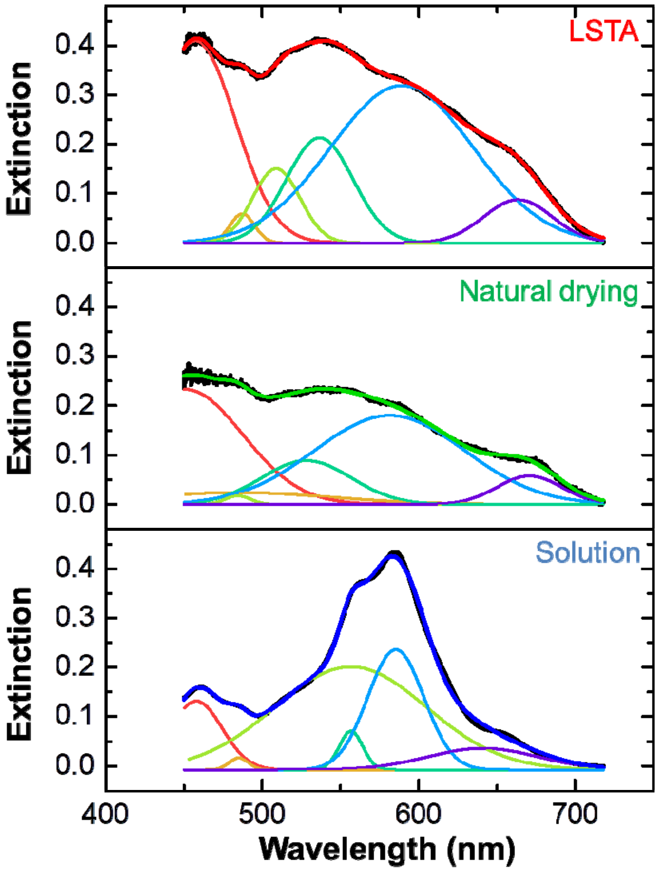

Supplementary Figure S3 | Peak analysis of extinction spectra for LSTA-produced structures, Natural drying-produced structures, and diporphyrin dispersed in toluene.

Supplementary Table S1 | Peak wavelengths obtained by peak analysis in Figure S3.

	Peak wavelength (nm)				
**LSTA**	**458**	**487**	**509&537**	**589**	**663**
**Natural drying**	**451**	**484**	**528**	**581**	**671**
**Solution**	**458**	**485**	**556**	**585**	**641**

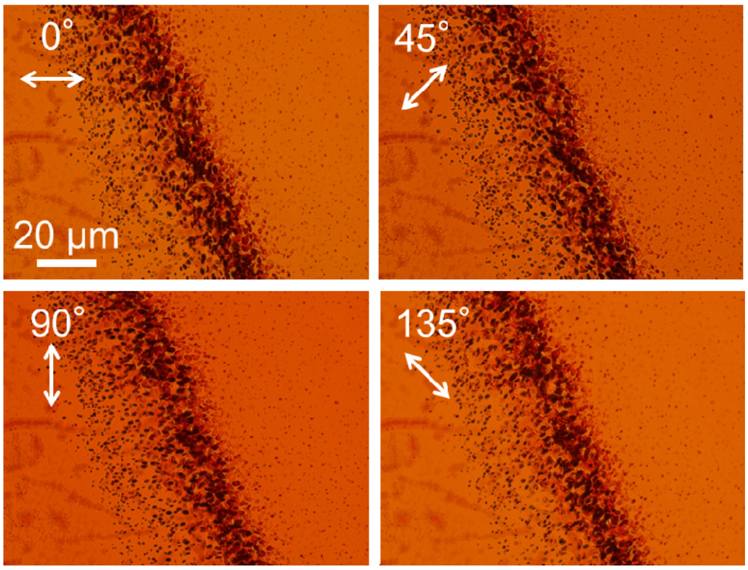

Supplementary Figure S4 | Optical transmission images of natural drying-produced structures at various polarisation angles.

Supplementary Table S2 | Intensity at peak wavenumber of each structure.

		Peak wavenumber (cm^{-1})			
		~1120	~1515	~1575	~1615
Intensity	LSTA region <i>	576	375	705	574
	LSTA region <ii>	1319	567	754	1203
	Natural drying	1052	409	845	951
